# Cancer computational biology

**DOI:** 10.1186/1471-2105-12-120

**Published:** 2011-04-26

**Authors:** Zohar Yakhini, Igor Jurisica

**Affiliations:** 1 Agilent Laboratories, Tel-Aviv, Israel; 2 Computer Science Department, Technion, Haifa, Israel; 3 Ontario Cancer Institute, PMH/UHN and the Campbell Family Institute for Cancer Research, IBM Life Sciences Discovery Centre, Toronto, Ontario, Canada; 4 Department of Computer Science, University of Toronto, Toronto, Ontario, Canada; 5 Department of Medical Biophysics, University of Toronto, Toronto, Ontario, Canada

## Editorial

Introduction of high-throughput measurement technologies combined with the increase of the scientific knowledge base, with respect to our understanding of cellular and biological processes, resulted in establishing computer and information science as an important and fundamental component of modern biology. High-throughput measurement technologies, such as microarray-based profiling, mass spectrometry screens, and high-throughput sequencing, give rise to several computational challenges. On one hand, they require a rigorous approach to assay design. Scientists and technology developers work on optimizing assay components so as to maximize the information obtained through the measurement. On the other hand, the use of high-throughput measurement gives rise to large quantities of data that needs to be pre-processed and analyzed to obtain meaningful knowledge. This processing and analysis is performed on various levels - from pre-processing the raw data, such as images from microarrays or raw sequence reads - to analyzing the data and to the discovery of biomarkers or other biologically meaningful characteristics. Measurement technology addresses several aspects of cellular processes such as DNA, RNA, proteomics, metabolomics, epigenetics and pathways. This increase in the scientific knowledge base also leads to a central role played by data analysis and modeling, strongly grounded in computational methods. Systems biology or integrative biology approaches and network analysis are of specific importance in this context.

The above is even further emphasized in the context of cancer research. Samples are complex and heterogeneous, and cancer related mechanisms involve many layers of the process that leads from the genome to cellular function. One example of a specific need of cancer is the study of large scale aberrations in the genome. CNVs (copy number variations) were recently recognized as abundant in normal cell populations and as related to many other disease types but they are still a hallmark of cancer [1,2]. Genomes in cancer cells often have a structure that allows them to bypass growth control cellular processes. Regions coding for tumor suppressor genes are often deleted and regions harboring oncogenes may be amplified. This is the case, for example, for p16 and myc, respectively [3-5]. Rearrangements, such as inversions and translocations, give rise to tumor-driving fusion products as in the case of BCR-Abl and the Philadelphia Chromosome as well as in more recent findings implicating fusion structures in solid tumors. Cancer research therefore makes use of data analysis methods and tools that address interpretation of copy number data and the understanding of the effect of genome changes on transcriptome level as well as proteome level profiles of tumors. Other specific computational needs of cancer research are related to epigenetic changes, somatic evolution, definition of gene sets in the context of specific cancer types, and to drugs and data that measures the effects of drugs.

**Figure 1:**
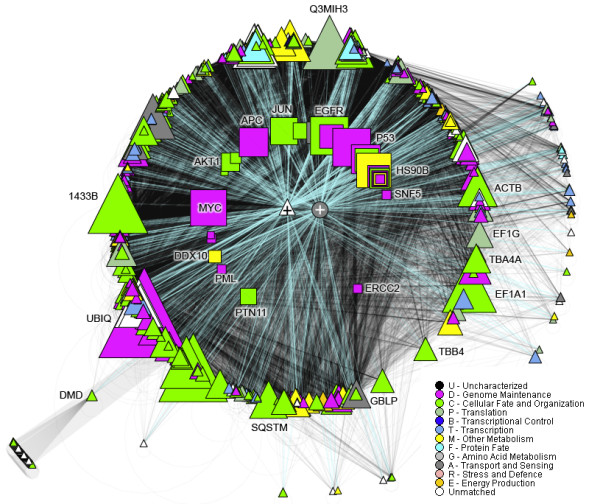
**Human interactome from I2D ver. 1.9 **http://ophid.utoronto.ca/i2d** - 278,214 physical protein-protein interaction (of which 158,549 are unique), connecting 14,641 proteins**. Using concentric circle layout, Sanger CENSUS cancer genes are used as a root (523 proteins connected directly by 1,180 interactions), and proteins with fewer than 51 interacting partners are collapsed in the two central points (to reduce number of objects and make the resulting SVG file editable in Adobe Illustrator). Node size corresponds to node degree, and node color corresponds to GeneOntology biological function. Visualization was done in NAViGaTOR ver. 2.2.1 http://ophid.utoronto.ca/navigator.

Computational biologists focusing on cancer develop methods for the genome scale characterization of tumors, on various levels of the molecular process. Data analysis methods often rely on the analysis of high-throughput measurement data and they provide understanding of the relationship between various molecular characteristics of cells. For example - how do genome structural aberrations and changes in copy number, a result of increased genome instability in cancer, affect the expression of genes and other functional elements such as miRNA, and how do the latter changes affect the function of related proteins. Understanding of the association of genomic characteristics and clinical properties of primary tumor samples, xenografts or cell lines contributes to personalized cancer medicine through the development of predictive biomarkers of drug efficacy. Many research projects therefore aim to discover biomarkers, at either genome, transcriptome or proteome level that are prognostic of cancer progression or predictive of response to specific therapeutic agents [6,7]. Cancer computational biology also focuses on analyzing molecules and processes that play a major role in cancer. An example is the analysis of cell cycle regulatory proteins and of immune response elements through the use of mathematical network and correlation models (For example - [8]). Many resources, such as IMEx [9], I2D [10], KEGG [11], PathwaysCommons [12], Reactome [13], i-HOP [14], STRING [15], GeneCards [16], mirDIP [17] and tools like Cytoscape [18], GSEA [19], NAViGaTOR [20] and GOrilla [21] provide some of the necessary bioinformatics infrastructure for integrative cancer research. Figure 1 exemplifies the integration of a cancer gene list from Sanger CENSUS data, highlighted within the human protein-protein interaction network.

### The meeting and the papers in this collection

RECOMB Cancer Computational Biology (RCCB) is a RECOMB satellite workshop that focuses on computational, statistical and algorithmic questions related to cancer. RCCB 2010 http://bioinfo.cs.technion.ac.il/people/zohar/recombccb2010/ took place in Oslo, Norway, adjacent to the biannual meeting of the European Association for Cancer Research (EACR). This meeting followed the first RCCB, which was held in San Diego in 2007. In 2011 RCCB will be held in conjunction with the main RECOMB conference in Vancouver; http://compbio.cs.sfu.ca/recomb2011/satellite/.

Integrative computational biology in cancer research benefits from close collaboration among diverse disciplines - computational biology, cancer research, genomics, proteomics to name a few. This collaboration also manifested itself in the meeting, bringing together speakers and attendees from various disciplines. The full program and to video recordings of many of the talks are listed on the meeting's website http://bioinfo.cs.technion.ac.il/people/zohar/recombccb2010/program.html. We will highlight three of the thematic foci of the meeting:

• **Cancer and copy number instability**. Chromosomal instability and changes in copy number are amongst the most important hallmarks of cancer. In many cases copy number differences drive differences in clinical behavior and in susceptibility to treatments. The understanding of the structure of chromosomal aberrations in cancer requires accurate measurement technologies coupled with data analysis and interpretation tools. Chris Greenman, from Wellcome Trust Sanger Institute, described an algorithmic approach to studying the evolution of genomic instability in cancer, through the analysis of sequencing data. In particular these methods attempt to infer orders of occurrence for observed aberrations, both mutations and copy number changes. Peter van Loo, from KU Leuven, described ASCAT - a tool for analyzing allele-specific copy number data, and discussed some results obtained for a breast cancer cohort. These include differences between various breast cancer subtypes. Basal-like samples were found to have significantly higher frequency of LOH compared to the other types. Hiroko Solvang, from Oslo University Hospital, talked about the relationship between copy number and expression levels [22]. Yinyin Yuan, from Cambridge University, described an approach to studying the regulation relationship between copy number and expression when focusing on specific disease subtypes, e.g., ER-positive and ER-negative samples. Finally, Anna Ritz, from the Computer Science Department in Brown University, described an algorithmic approach to determining genomic structure from high-throughput copy number data [23]. This approach considers recurrent events in datasets with multiple samples. While other approaches study common aberrations, the Ritz et al. approach identifies recurrent breakpoints, and thereby infers potential fusion and gene truncation events. Such events are obviously extremely relevant to cancer pathogenesis and development processes, and the Ritz et al. method enables an analysis that may lead to significant findings.

• **Systems biology approach to cancer**. Three keynote talks addressed studies that consider the full system level properties of cancer and what can be learned from them. Joe Gray's Lab, at LBNL, worked with 50+ breast cancer cell lines and ~80 different compounds targeting the ERBB2 pathway, generating IC50 data for these combinations and inferring genomic determinants of treatment efficiency. Yossi Yarden's Lab, at the Weizmann Institute, studied the response to EGF stimulus, measuring both the transcriptome reaction and a comprehensive miRNA reaction. A talk at the meeting described some of these results in the context of previous studies of the EGF pathway. Special emphasis was put on miRNAs that are immediately down-regulated in response to the signal. Israel Steinfeld, from the Technion in Haifa, described a breast cancer study integrating miRNA and mRNA profiles that further expands our understanding of miRNA activity in cancer. This includes the association of processes such as proliferation and cell cycle to the activity of miRNAs and their target genes. High-throughput proteomics profiling, including the understanding of activation status, can be an important piece in the cancer system biology picture, including implications for direct targeted therapy. The realization of this potential requires accurate and high-throughput proteomics measurement technologies as well as data analysis techniques to support interpretation and inference. Gordon Mills, from MD Anderson Cancer Centre, described the ongoing work he and colleagues are doing on developing and optimizing Reverse Phase Protein Arrays (RPPAs), and on using them in cancer-related studies.

• **Networks**. Understanding the relationship between various molecular elements can extend the understanding of larger scale processes and thereby expand the repertoire of molecules related to cancer. Anaise Baudot, from CNIO (Centro Nacional de Investigaciones Oncológicas), presented her joint work with Enrico Glaab and others on extending functional annotation networks using information derived from protein-protein interaction networks [24]. They map proteins annotated for different cellular processes onto a large protein-protein interaction networks and attempt to extend these processes by adding the most densely interconnected network partners, when said connectivity exceed certain threshold criteria. Gurkan Bebek, Case Western Reserve University, described a process that enables the prioritization of various mutation states in a key gene, according to their effect on protein levels, in specific contexts, most notable in the context of a specific cancer type. The performance of the software, called PETALS, was demonstrated through the analysis of APC as a driver of colon cancer [25].

The conference offered a full paper track, and in this thematic collection we present the papers that were presented as talks in Oslo and were also selected, through an additional co-ordinated review process, to be published by *BMC Bioinformatics *[22-25].
